# Cardiac metabolomics and autopsy in a patient with early diffuse systemic sclerosis presenting with dyspnea: a case report

**DOI:** 10.1186/s13256-015-0587-7

**Published:** 2015-06-10

**Authors:** Tracy M Frech, Monica P Revelo, John J Ryan, Ami A Shah, Jessica Gordon, Robyn Domsic, Faye Hant, Shervin Assassi, Victoria K Shanmugam, Monique Hinchcliff, Virginia Steen, Dinesh Khanna, Elana J Bernstein, James Cox, Nick Luem, Stavros Drakos

**Affiliations:** 1 Department of Internal Medicine, University of Utah and Veterans Affair Medical Center, Salt Lake City, UT USA; 2 University of Utah Department of Pathology, Salt Lake City, UT USA; 3 Department of Medicine, Division of Cardiovascular Medicine, University of Utah, Salt Lake City, UT USA; 4 Johns Hopkins University School of Medicine, Baltimore, MD USA; 5 Hospital for Special Surgery, New York, NY USA; 6 University of Pittsburgh, Pittsburgh, PA USA; 7 Medical University of South Carolina, Charleston, SC USA; 8 University of Texas, Houston, TX USA; 9 George Washington University, Washington, DC USA; 10 Northwestern University, Chicago, IL USA; 11 Georgetown University, Washington, DC USA; 12 University of Michigan, Ann Arbor, MI USA; 13 Columbia University, New York, NY USA; 14 Departments of Biochemistry and Metabolomics Core Facility, University of Utah School of Medicine, HSC Cores, Salt Lake City, UT USA

**Keywords:** Autopsy, Metabolomics, Scleroderma, Systemic sclerosis

## Abstract

**Introduction:**

Diffuse systemic sclerosis is associated with high mortality; however, the pathogenesis of cardiac death in these patients is not clear.

**Case presentation:**

A 56-year-old Caucasian female patient presented with dyspnea and requested to donate her body to science in order to improve understanding of diffuse systemic sclerosis pathogenesis. She had extensive testing for dyspnea including pulmonary function tests, an echocardiogram, cardiac magnetic resonance imaging, and right heart catheterization to characterize her condition. Her case highlights the morbidity seen in this disease, including the presence of extensive skin thickening, digital ulcerations, and scleroderma renal crisis.

**Conclusion:**

In this case report, we present the finding of cardiac tissue metabolomics, which may indicate a problem with vasodilation as a contributor to cardiac death in diffuse systemic sclerosis. The use of autopsy and tissue metabolomics in rare disease may help clarify disease pathogenesis.

## Introduction

Diffuse systemic sclerosis (SSc) is associated with a high morbidity and mortality, and collaborative efforts are needed to understand its pathogenesis [1]. The Prospective Registry of Early Systemic Sclerosis (PRESS), a multicenter incident cohort study of patients with early diffuse cutaneous SSc, has the goal of advancing the understanding of disease pathogenesis and identifying novel biomarkers [2]. The PRESS investigators elected to study patients with recently diagnosed diffuse SSc initiated on appropriate treatment to examine predictors of these patients’ morbidity and mortality. We present a case report of a PRESS patient who requested to donate her body to science to highlight the importance of using autopsy to better understand mortality in this rare disease. This is the first case report of using cardiac tissue metabolomics in diffuse SSc to better understand disease pathogenesis.

## Case presentation

A 56-year-old Caucasian woman with a 20-year history of type II diabetes mellitus presented with increased fatigue, bilateral lower extremity edema, and tightness of her skin in her lower extremities in 2012. Her primary care physician evaluated her cellular blood counts, chemistries, and thyroid-stimulating hormone, and obtained an echocardiogram. All of these studies were normal. A sleep study revealed mild obstructive sleep apnea, which was treated.

Within six months, in addition to lower extremity skin thickening, she developed dyspnea, Raynaud’s phenomenon, and digital ulcerations. She was referred to a rheumatologist at that time and found to be positive for anti-nuclear antibodies, at 1:640 in a nucleolar pattern, and RNA polymerase III antibody. Tests for other SSc antibodies, including anti-centromere, anti-fibrillin, anti-topoisomerase, and anti-Th/To, were negative. Her blood pressure and creatinine were noted to be normal. Her hemoglobin A1c was 7.6%. She was not on an angiotensin-converting enzyme inhibitor (ACE-I).

At a rheumatology appointment six months after her initial symptoms started, pulmonary function tests demonstrated an isolated decrease in diffusing capacity (38% predicted). A repeat echocardiogram suggested the interim development of moderate pulmonary hypertension with an estimated right ventricular systolic pressure of 45mmHg and an impaired relaxation pattern of left ventricular filling. A computed tomography scan of her chest did not reveal the presence of a pulmonary embolism, and there was no evidence of interstitial lung disease. Cardiac magnetic resonance imaging (MRI) did not show any delayed enhancement that would suggest prior myocardial injury or infarction. The images revealed that she had normal stress and resting perfusion, as well as hyperdynamic left ventricular function with an ejection fraction of 77% and no wall motion abnormalities. However, there was mild bi-atrial enlargement and her right ventricle was dilated with normal right ventricular systolic function. A right heart catheterization established the diagnosis of pulmonary hypertension, with right atrial pressure of 12mmHg, right ventricular pressure of 53/17mmHg, pulmonary arterial (PA) pressure of 45/16mmHg (mean, 33mmHg), wedge pressure of 20mmHg, cardiac output of 7L/min, cardiac index of 3.5L/min, and peripheral vascular resistance of 2 Wood units. She was noted to be anemic with a hemoglobin level of 7.4g/dL.

Based on her cardiac catheterization results, she was treated with diuresis and a phosphodiesterase 5 inhibitor. At her one-month follow-up visit after the cardiac catheterization, her digital ulcerations were healing and lower extremity edema was improved, but her modified Rodnan skin score had worsened by six points (27 to 33). Methotrexate was prescribed, but our patient reported intolerable nausea after two doses. Owing to the anemia, she had an upper endoscopy. This led to a diagnosis of gastric antral vascular ectasia that was treated with argon plasma coagulation. Our patient was hesitant to continue methotrexate, thus mycophenolate mofetil was prescribed. Our patient did not receive any steroid therapy.

One year after her initial symptom of SSc, she had a scleroderma renal crisis (SRC), with a presenting blood pressure of 178/76mmHg and creatinine level of 1.77mg/dL. Microangiopathic hemolytic anemia was not present. Our patient was hospitalized and her blood pressure was controlled with an ACE-I. Our patient attended follow-up visits every other month for the next four months. Despite well-controlled blood pressure; echocardiogram improvement (normal ejection fraction); resolution of anemia following treatment for gastric antral vascular ectasia; creatinine stabilization (1.44mg/dL); and modified Rodnan skin score stabilization (maximum 38), our patient reported progressive fatigue and weakness. An electromyogram, nerve conduction studies, and levels of muscle enzymes were normal, and physical therapy was prescribed. She reported mild improvement in all symptoms at a follow-up visit two months later.

One year after her diagnosis of SSc and two months after her last clinic appointment, our patient was hospitalized for acute onset left-sided heart failure, with an ejection fraction of 20% documented by echocardiogram. An ECG revealed that her sinus rhythm and cardiac enzyme levels were normal. A repeat right and left heart catheterization showed a right atrial pressure of 19mmHg, right ventricular pressure of 64/7mmHg, PA pressure of 69/27mmHg (mean, 39mmHg), wedge pressure of 19mmHg, cardiac output of 6.8L/min, cardiac index of 3.3L/min, and only mild luminal abnormalities in her right coronary and left circumflex arteries. During this hospitalization, our patient stated that if she were to die, she wished to donate her body to science to help further physician understanding of SSc. One day after making this statement, she died of pulseless electrical activity, despite being followed closely at a clinic dedicated to SSc care and having no limitations to access.

**Figure 1:**
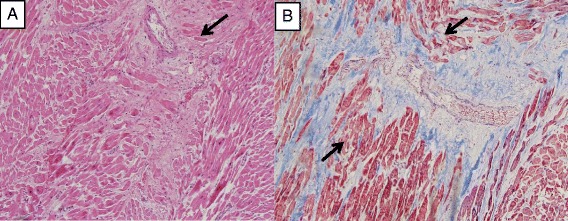
**(A)** Hematoxylin and eosin-stained section shows myocardial tissue with interstitial fibrosis. There is focal myocyte hypertrophy. Inflammation is not identified. Small artery (arrow) does not show abnormalities. **(B)** On trichrome stain, the interstitial fibrosis is highlighted in blue and myofibrillar loss (arrows) is also seen.

An autopsy was immediately performed. She had cardiac enlargement, right and left ventricular chamber dilatation, pleural effusions, hepatosplenomegaly, and ascites indicative of heart failure. Atherosclerosis of her right coronary artery with 20% occlusion of the lumen was identified. Sections of her right and left ventricles, and septum showed focal interstitial fibrosis (Figure 1). There was mild perivascular fibrosis in her left ventricle. Trichrome staining confirmed the presence of collagen; there were no other microvascular changes. There was myocyte hypertrophy, but no signs of inflammation. Her lungs showed moderate interstitial fibrosis, septal thickening, and large arteries with intima fibrosis. Segments of her esophagus, stomach, small bowel, and large bowel showed marked collagen deposition in the lamina propria. There was moderate autolysis without significant inflammation. Sections of her kidney showed several globally sclerotic glomeruli, moderate fibrous arterial intimal thickening, and interstitial fibrosis. There were no inflammatory infiltrates, casts, or crystals. There was glomerular enlargement, nodular increases in mesangial matrix, and capillary wall thickening consistent with nodular diabetic glomerulosclerosis. No thrombotic microangiopathy of the small vessels was noted. Her bone marrow was hypercellular for age but showed trilineage hematopoiesis with an approximately normal ratio of myeloid to erythroid precursors. No other significant abnormalities were reported. The pathologist reported her likely cause of death as a fatal dysrhythmia due to myocardial fibrosis and atherosclerosis.

Metabolomics were used to investigate which metabolism and signaling pathways played a key role in her heart failure. For this investigation, the heart was immediately placed in a −80°C freezer. Subsequent quantitative analysis of a large number of small metabolites (<1000Da) within a cell or tissue and principle component analysis allow maximum variation in the data to be described, providing a fingerprint of physiological and pathophysiological status. In our study, the comparative analyses of metabolomes were performed by a combined approach of gas chromatography–mass spectrometry spectroscopic techniques with subsequent computer analysis of the data generated. Our patient’s tissue was compared to averages of 79 metabolites in 16 other patients who suffered cardiac death. In our patient, the essential amino acids (2-aminoadipic acid, lysine, valine, leucine, alanine, glutamic acid, proline, aspartic acid, asparagine, methionine, cysteine, tyrosine, and histidine) were the most elevated metabolic class. Our patient’s lipid, peptide, and carbohydrate metabolites were similar to those in the other 16 patients tested. Of interest, despite her history of SRC, our patient’s creatinine level was lower than any of the other samples tested.

## Discussion

The index case presented here is of a PRESS patient at high risk for morbidity and mortality, who stated a desire to donate her body to science. As per this patient’s desire, the PRESS investigators acknowledge that a more objective and precise system to identify specific cause of death is needed in rare diseases to better understand their pathogenesis. This patient was followed closely at a dedicated SSc clinic, thus her care was guideline based [2,3]. Nonetheless, the outcome was poor and, despite appropriate therapeutics, she died suddenly. In SSc patients, myocardial fibrosis, conduction system disease, and autonomic neuropathy are substrates predisposing to supraventricular and ventricular arrhythmias, which in some individuals can cause sudden death. The use of electroanatomical mapping may have a role in these patients to reveal possible arrhythmogenic foci [4]. While there are contradictory reports regarding the prevalence of atherosclerosis in SSc, it is possible that, in our patient, her longstanding diabetes contributed to her adverse cardiac outcome [5]. Once cardiac dysfunction becomes apparent in patients with SSc it can severely increase morbidity and rapidly lead to death, such as seen in this case. The etiology of cardiac fibrosis may be repeat ischemia and reperfusion injury [6].

The prevalence of primary cardiac involvement in SSc may be difficult to determine owing to the numerous possible cardiac manifestations in a phenotypically diverse patient population. Histologic studies, which reveal the presence of myocardial involvement, often disagree with clinical studies [5]. In our patient, we determined that metabolomics may provide further information on her presumed cardiac death. Metabolomics is a powerful tool to map perturbations in the metabolic system and enables simultaneous quantification of several metabolites that might provide insights into disease pathogenesis [7]. We used metabolomics to provide a systematic study of the polar metabolites (metabolome) present in the cardiomyocyte at the time of her death. There are reported end-point metabolites of biological events after cardiac arrest and resuscitation [8]. In this pilot study, the metabolomics approach offered insight into the cardiac molecule regulation and signaling at the time of our patient’s presumed cardiac death. The metabolome represents the complete set of low molecular weight (typically <1500Da) metabolites produced by an organism, which are the end products of gene expression, and reflects the physiological state of a biological system [9]. Because untargeted metabolomics essentially provides a snapshot of the genome in its interaction with the environment, it is useful in biomarker discovery, diagnostics, and may reveal specific metabolic patterns of disease [9]. In our patient with diffuse SSc, the primary finding was that her levels of essential amino acids were elevated. While no statistical significance could be assigned to these results because they were from one biological replicate, it is an interesting finding nonetheless. The dibasic amino acids share plasma membrane transporters with arginine, a rate-limiting substrate for nitric oxide synthase, which is a critical mediator of vasodilation [10]. Our patient’s profile was different from the metabolomic network of seven metabolites reported to be associated with death (gamma-glutamylphenylalanine, gamma-glutamyltyrosine, 1-arachidonoyl GPC(20:4), taurochenodeoxycholate, 3-(4-hydroxyphenyl) lactate, sucrose, kynurenine) in patients studied in an intensive care unit with systemic inflammatory response criteria [11].

Historically, autopsy analysis was instrumental for understanding SSc pathogenesis [12]. Previous autopsy series of cardiac involvement in SSc describe hypertrophy and/or fibrosis primarily of the myocardium; however, fibrosis of the endocardium, pericardium, and epicardium has been reported [13,14]. These autopsy studies suggest that cardiac fibrosis represents a late feature of disease; however, cardiac remodeling, characterized by myocardial fibrosis, particularly within the interstitium in patients with SSc, can be identified on endomyocardial biopsy before any signs or symptoms of heart failure appear [15]. Thus, another important aspect of this early diffuse SSc case is that left ventricle changes were comparable to the right ventricle changes at autopsy. This finding highlights that patients with diffuse SSc may have distinct changes in both the right and left ventricles, independent of the presence of PAH or heart failure. In fact, the use of cardiac MRI in SSc, which can show delayed enhancement consistent with fibrosis, demonstrates that cardiac involvement is likely more common than previously recognized on echocardiogram [16]. The importance of a better understanding of low-grade endocardial and myocardial inflammation and fibrosis in patients with diffuse SSc is paramount for guiding therapy and predicting outcomes [17]. In particular, the role of ACE-I in diffuse SSc must be clarified because it may mask SRC [18], yet, as highlighted by this case, may have a role in cardiac care. Furthermore, this case highlights a need for renewed attention on the utility of autopsy to enhance our understanding of diffuse SSc pathogenesis on a molecular level. A delayed-enhancement cardiac MRI failed to identify myocardial fibrosis that was present on a histological assessment of the myocardium following autopsy. Thus, post-mortem studies may enhance our knowledge of visceral involvement in diffuse SSc.

## Conclusions

This case highlights the importance of autopsy. Our patient suffered from the many effects of SSc and requested that her body be donated to science to help further an understanding of the pathogenesis of SSc. It is likely that the pace of discovery will dramatically accelerate in SSc research as increasingly comprehensive screening of the genome, transcriptome, proteome, and metabolome are applied. To optimize these new technologies, however, it is critical that autopsy is considered in patients with SSc to allow tissues for research to be methodically collected and processed. The PRESS investigators acknowledge that autopsy holds immense potential for understanding mechanisms of disease and facilitating the development of diagnostics, and perhaps personalized therapeutic strategies, in SSc.

## Consent

This patient consented to participate in the PRESS registry and its publications. She was committed to the discovery of pathogenesis in SSc. Written informed consent was obtained from the patient's next-of-kin for publication of this case report and any accompanying images. A copy of the written consent is available for review by the Editor-in-Chief of this journal.
